# Fexuprazan for upper gastrointestinal protection in patients with ischemic stroke and gastroesophageal reflux disease (GERD) receiving antithrombotic therapy: A preliminary prospective single-arm observational Study

**DOI:** 10.1371/journal.pone.0346145

**Published:** 2026-04-03

**Authors:** Minyoul Baik, Jeong A. Shin, Joonsang Yoo, Jinkwon Kim

**Affiliations:** Department of Neurology, Yongin Severance Hospital, Yonsei University College of Medicine, Yongin-si, Gyeonggi-do, South Korea; Dr. Dayaram Patel Pharmacy College, INDIA

## Abstract

**Background:**

Proton pump inhibitors are standard for preventing upper gastrointestinal (UGI) complications in patients receiving antithrombotic therapy, but their limitations highlight the need for alternatives. Potassium-competitive acid blockers (P-CABs), such as fexuprazan, are emerging substitutes, yet evidence supporting their protective role in this setting remains limited. This study aimed to evaluate the effect of fexuprazan in patients with acute ischemic stroke (AIS) and gastroesophageal reflux disease (GERD) receiving antithrombotic therapy.

**Methods:**

This prospective, single-center, single-arm study included consecutive patients with AIS and GERD who were receiving antithrombotic therapy and treated with fexuprazan between June 2023 and March 2025. At the study hospital, all admitted AIS patients were systematically screened for GERD using the Gastroesophageal Reflux Disease Questionnaire (GerdQ), with a score ≥8 indicating GERD. The primary outcome was the change in GerdQ score from baseline to 12 weeks after the index AIS. Safety outcomes included recurrent stroke or serious adverse events within 12 weeks. The secondary efficacy outcome was the occurrence of a composite of UGI clinical events.

**Results:**

Among 623 patients with AIS screened during the study period, 18 patients with GERD receiving antithrombotic therapy and treated with fexuprazan were enrolled and followed for 12 weeks. The mean age was 65.6 years, and 9 patients (50.0%) were male. The mean GerdQ score significantly improved from 8.8 ± 2.3 at baseline to 6.6 ± 1.7 at 12 weeks (*P* = 0.003). A normal GerdQ score (<8) was achieved in 88.9% of the patients. One patient (5.6%) experienced a UGI clinical event and one had a recurrent transient ischemic attack.

**Conclusions:**

This preliminary prospective single-arm study using a structured symptom-based assessment suggests that fexuprazan may be associated with symptom relief in patients with AIS and GERD receiving antithrombotic therapy. However, due to the small sample size and lack of a control group, these findings should be interpreted as hypothesis-generating and warrant further confirmation in larger, controlled trials.

## Introduction

Acute ischemic stroke (AIS) remains a major health burden, with a 1-year stroke recurrence rate of 5.1% and mortality of 9.6% in 2020 in South Korea [1]. To prevent recurrent stroke, antithrombotic therapy is a cornerstone treatment [2]; however, it carries a risk of exacerbating gastroesophageal reflux disease (GERD) and may lead to upper gastrointestinal (UGI) bleeding [3,4]. GERD is a condition in which gastric contents reflux into the esophagus or beyond and causes troublesome symptoms and complications that interfere with daily life [5] and increases the risk of UGI bleeding [6]. Although clinical guidelines recommend the use of proton-pump inhibitors (PPIs) to alleviate GERD symptoms and reduce UGI bleeding risks in high-risk patients receiving antithrombotic therapy, unmet needs remain [3,4].

P-CABs, a novel class of acid-suppressive agents, competitively bind to the potassium site of the gastric proton pump (H ⁺ /K ⁺ -ATPase), allowing for rapid, potent, and sustained acid inhibition compared with conventional PPIs [7–10]. Because of their acid stability, minimal CYP2C19 interaction, and more consistent pharmacodynamic effects, P-CABs may offer advantages over PPIs in treating acid-related diseases, including GERD and preventing UGI bleeding in patients receiving antithrombotic therapy [7–10]. Fexuprazan (Daewoong Pharma, 2022), a P-CAB, has demonstrated non-inferiority to esomeprazole in the treatment of GERD [11,12]. However, clinical evidence remains limited regarding the protective effect of P-CABs, including fexuprazan, against UGI complications in patients receiving antithrombotic therapy.

This study aimed to explore the potential effect of fexuprazan in patients with AIS and GERD treated with antithrombotic therapy, focusing on the prevention of aggravation of GERD symptoms after 12 weeks, as assessed using the Gastroesophageal Reflux Disease Questionnaire (GerdQ), and the occurrence of UGI events.

## Materials and methods

### Study population and design

This was a prospective, single-center, single-arm, observational cohort study conducted at Yongin Severance Hospital, South Korea. This study was approved by the Institutional Review Board of the Yongin Severance Hospital, Yonsei University Health System (approval no. 9-2024-0002). Written informed consent was obtained from all participants or their legally authorized representatives prior to enrollment.

Patients with AIS admitted to the center between June 2023 and March 2025 were screened consecutively. Patients were enrolled if they met the following inclusion criteria: (1) age over 18 years; (2) AIS admitted within 1 week of symptom onset, with lesions confirmed by brain computed tomography or magnetic resonance imaging; (3) GERD, defined as a Korean version of GerdQ score ≥ 8 or a documented history of GERD; (4) prescription of antithrombotics; and (5) prescription of fexuprazan. Patients were excluded if they met any of the following criteria: (1) allergy to fexuprazan, (2) inability to complete the GerdQ, (3) pregnancy or lactation, or (4) any condition that, in the judgment of the clinician, rendered the patient inappropriate for enrollment in the study.

As this was a preliminary exploratory study, the sample size required to demonstrate a representative effect of fexuprazan on GerdQ score differences after 12 weeks of treatment in patients with AIS receiving antithrombotic therapy could not be estimated due to the lack of previous literature. The authors initially planned to enroll 50 patients, considering the recruitment period and available study funds; however, the target number was reduced to 20 patients because of the slow enrollment rate.

### Procedures and gastroesophageal reflux disease questionnaire

Baseline clinical data, including age, sex, history of hypertension, diabetes mellitus, GERD, body mass index, and current smoking and alcohol use status were collected. All patients were evaluated and treated according to standard stroke guidelines [2]. At the study hospital, all patients with AIS were screened for high risk of GERD or UGI bleeding during admission using the GerdQ, except for those who were unable to complete the questionnaire owing to clinical conditions such as decreased mental status or aphasia.

The GerdQ is a validated six-item tool with a score ≥8 indicating GERD, and has shown good diagnostic performance across populations [13,14], including a high positive predictive value (88.1%) in the Korean version [15]. The GerdQ consists of four positive predictors (heartburn, regurgitation, sleep disturbance, and use of over-the-counter medications) and two negative predictors (epigastric pain and nausea), each scored based on symptom frequency over the past week. Positive items were scored 0–3, and negative items were reverse-scored 3–0, yielding a total score range of 0–18. To screen for GERD, patients completed the initial GerdQ based on symptoms experienced the week before AIS-related admission.

### Clinical outcomes

The primary efficacy outcome was GerdQ score measured 12 weeks after the index AIS. Safety outcomes included recurrent ischemic stroke or transient ischemic attack, serious adverse events, or all-cause death within 12 weeks after the index AIS. The secondary efficacy outcome was occurrence of a composite of UGI clinical events: overt bleeding of gastroduodenal origin (confirmed by means of upper endoscopy or radiography), overt UGI bleeding of unknown origin, bleeding of presumed occult gastrointestinal origin with a documented decrease in hemoglobin of 2 g per deciliter or more or in the hematocrit by 10% or more from the baseline value, symptomatic uncomplicated gastroduodenal ulcer (confirmed by means of endoscopy or radiography), persistent pain of presumed gastrointestinal origin with a duration of 3 days or more and with gastroduodenal erosions (confirmed by means of endoscopy), obstruction, or perforation, and administration of a PPI, either oral or intravenous, based on the treating physician’s clinical judgment [16].

### Statistical analyses

A paired t-test was used to compare the total GerdQ scores and each of the six questionnaire items at baseline and 12 weeks, as the Shapiro–Wilk test indicated the normal distribution of the paired differences. The mean difference and its 95% confidence interval (CI) were calculated for the primary outcome. Effect sizes were estimated using Cohen’s *d* for paired samples, along with corresponding 95% CIs [17]. Descriptive data regarding the outcomes of UGI clinical events, recurrent stroke or TIA, and all-cause death were described as frequencies and percentages. Statistical analyses were performed using the R software (version 3.5.1; R Foundation for Statistical Computing). Statistical significance was set at *P* < 0.05.

## Results

### Study population and baseline characteristics

Between June 2023 and March 2025, 623 patients admitted for AIS were screened. After excluding 605 patients based on the study criteria (S1 Fig in S1 File), 18 patients with AIS and GERD who were receiving both antithrombotic therapy and fexuprazan were included in the study. The mean age was 65.6 ± 11.7 years, and 9 (50.0%) were male (Table 1). The initial mean GerdQ score was 8.8 ± 2.3, and 10 patients (55.6%) had a previously documented history of GERD (Table 1).

**Table 1:** Characteristics of study participants.

Case	Age (years)	Sex	BMI	HTN	Diabetes mellitus	Current smoker	Alcohol use	GERD	GerdQ initial	GerdQ at 12 weeks	Antithrombotics at 12 weeks	Clinical outcomes
**1**	77	F	28.1	+	–	–	–	+	14	6	C	–
**2**	74	M	21.7	+	+	–	–	+	6	6	A + C	–
**3**	66	F	27.9	–	–	–	+	+	6	10	Edoxaban	–
**4**	74	F	19.2	–	+	–	–	+	6	6	C+Triflusal	–
**5**	60	M	26.5	+	–	+	+	+	8	6	A + C	–
**6**	66	M	28.6	+	–	–	+	+	11	6	A + C	–
**7**	61	F	23.7	–	–	–	–	+	10	6	C	–
**8**	56	F	25.6	+	+	–	–	–	8	6	A + C	–
**9**	76	F	20.0	–	–	–	–	+	6	6	C	–
**10**	68	M	21.7	+	+	–	–	+	6	6	Apixaban	–
**11**	76	F	30.5	+	+	–	–	–	9	6	A + C	TIA
**12**	53	M	26.8	+	–	+	+	–	10	6	A + C	–
**13**	74	M	25.6	+	–	–	+	+	12	6	A + C	–
**14**	54	M	26.7	+	–	–	+	–	8	6	Edoxaban	–
**15**	83	M	26.6	+	+	–	–	–	11	6	A + C	–
**16**	67	F	24.8	+	+	–	–	–	8	6	A + C	–
**17**	61	F	26.6	–	+	–	–	–	10	12	C	UGI event
**18**	34	M	27.1	–	–	+	+	–	9	6	A + C	–

A, aspirin; BMI, body mass index; C, clopidogrel; F, female; GERD, gastroesophageal reflux disease; GerdQ, Gastroesophageal Reflux Disease Questionnaire; HTN, hypertension; M, male; TIA, transient ischemic attack; UGI, upper gastrointestinal tract.

### Outcomes

**Fig 1:**
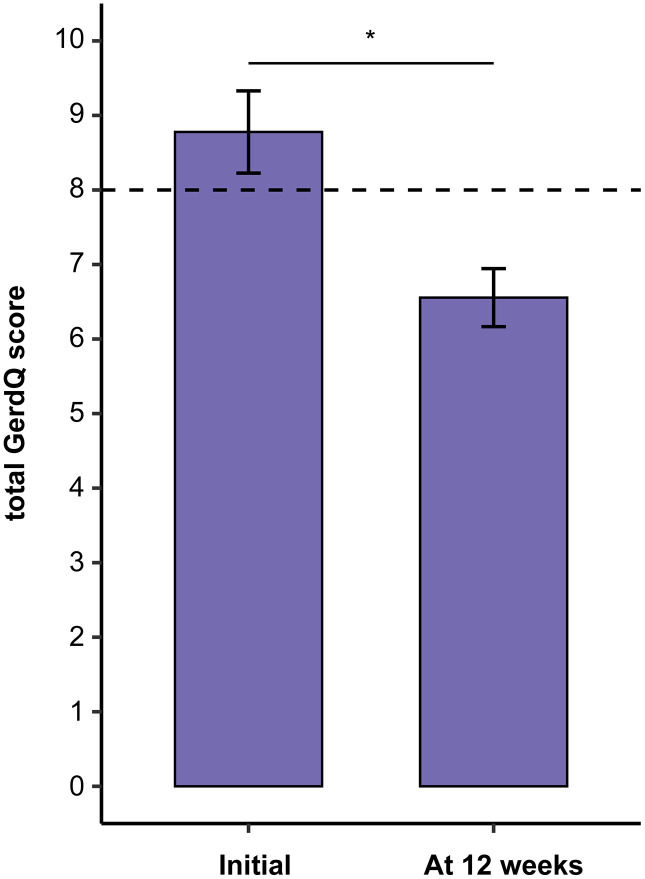
Change in total GerdQ score from initial visit and 12 weeks. Data are presented as mean ± standard deviation. Statistical significance was assessed using paired t-tests (^*^P < 0.05; ^**^P < 0.01; ^***^P < 0.001). GerdQ, Gastroesophageal Reflux Disease Questionnaire.

At the 12-week follow-up, the mean GerdQ score decreased from 8.8 ± 2.3 at baseline to 6.6 ± 1.7 (*P* = 0.003; Fig 1). The mean change in GerdQ score was −2.22 (95% CI, −3.69 to −0.76). The effect size of this change was large (Cohen’s *d* = 1.10; 95% CI, 0.22–1.98). A normal GerdQ score was observed in 16 (88.9%) patients (Table 1). At 12 weeks, 11 patients (61.1%) received dual-antiplatelet therapy (DAPT) (Table 1).

**Fig 2:**
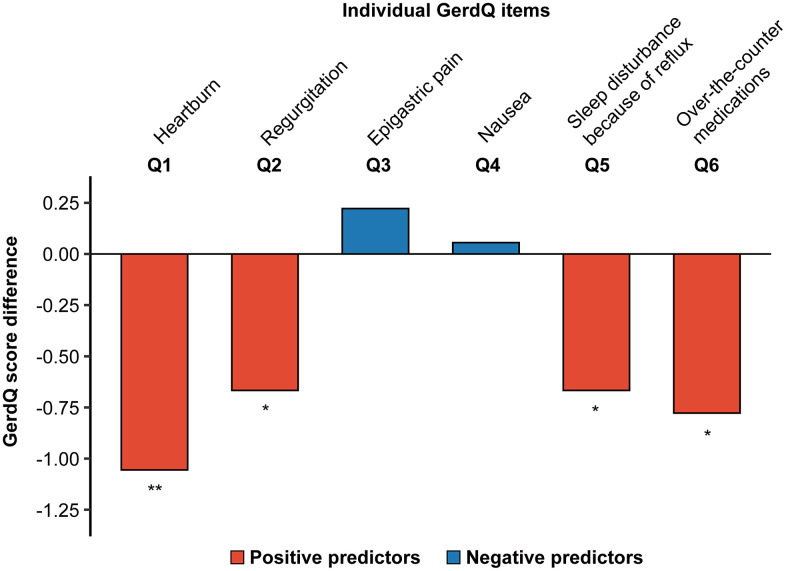
Changes in each GerdQ score from initial assessment to 12 weeks. The GerdQ includes four positive predictors of GERD: heartburn (Q1), regurgitation (Q2), sleep disturbance because of reflux symptoms (Q5), and use of over-the-counter medications (Q6), as well as two negative predictors: epigastric pain (Q3) and nausea (Q4). Statistical significance was assessed using paired t-tests (^*^P < 0.05; ^**^P < 0.01; ^***^P < 0.001). GerdQ, Gastroesophageal Reflux Disease Questionnaire.

The mean change in each questionnaire item indicated a favorable effect of fexuprazan, particularly in reducing positive predictor symptoms (all *P* < 0.05; Fig 2 and S1 Table in S1 File).

During the 12-week follow-up period, one patient (5.6%) experienced a recurrent transient ischemic attack. One patient (5.6%) experienced a UGI event, additional PPI therapy due to uncontrolled GERD symptoms, as evidenced by an increase in the GerdQ score from 10 to 12 at 12 weeks. Fexuprazan showed good overall tolerability, without discontinuation or serious adverse events.

## Discussion

In this study, we investigated the clinical outcomes associated with fexuprazan in patients with AIS and GERD receiving antithrombotic therapy. Fexuprazan was associated with improvement in GERD symptoms over the 12-week follow-up period, with approximately 90% of patients achieving a normal GerdQ score. These findings support the potential utility of fexuprazan in this population, although the results must be interpreted as preliminary given the small sample size and single-arm design.

P-CABs offer rapid and potent acid suppression with minimal interaction with CYP2C19, and favorable long-term tolerability [11,12], suggesting they may serve as an effective alternative to conventional PPIs. Although PPIs remain the standard therapy for gastroprotection in patients receiving antithrombotic treatment [18–21], concerns regarding potential adverse effects and drug interactions have contributed to their underuse [22]. Previous randomized trials have shown that fexuprazan is non-inferior to esomeprazole for GERD treatment [11,12]. Consistent with this, our preliminary observations suggest that fexuprazan may also alleviate GERD symptoms in patients with AIS receiving antithrombotic therapy.

Building on this body of evidence, P-CABs have also shown promising efficacy in preventing UGI bleeding. A previous randomized trial showed that vonoprazan, a P-CAB, was as effective as lansoprazole, a PPI, in preventing aspirin-associated ulcer recurrence [23]. In addition, it was associated with a significantly lower rate of UGI bleeding during the 24-week treatment period (0% vs. 2.9%) [23]. Similarly, our recent study using a Korean claim database also showed that P-CAB use was comparable to PPI use in preventing UGI bleeding in patients with percutaneous coronary intervention on DAPT [24]. Currently, an ongoing phase 4 randomized trial (NCT04416581) is comparing a P-CAB (tegoprazan) with a PPI (rabeprazole) for UGI protection in patients at high risk of UGI bleeding receiving antithrombotic therapy and is expected to provide high-quality evidence on the role of P-CABs in this setting [25].

In this context, our preliminary prospective study provides initial data suggesting the potential utility of fexuprazan in Korean patients with AIS and GERD receiving antithrombotic therapy, using a structured and quantifiable assessment tool. The mean GerdQ score decreased significantly over the 12-week treatment period with a large effect size (Cohen’s *d* = 1.10, exceeding the conventional threshold of 0.8) [17]. While this supports the clinical relevance of the observed symptom reduction, these findings must be interpreted with caution. Given the small sample size, absence of a control group, reliance on symptom-based assessment, and short follow-up duration, these observations should be interpreted strictly as hypothesis-generating. Notably, the observed improvement was primarily driven by a reduction in positive predictor items of the GerdQ, which may better reflect the severity of GERD symptoms [26]. If confirmed in larger studies, fexuprazan may provide clinically meaningful symptom relief that could assist in maintaining adherence to essential antithrombotic treatment [27]. Furthermore, considering that GERD is a risk factor for UGI bleeding in patients treated with antithrombotic therapy [6], this symptomatic improvement may imply a gastroprotective effect. However, because this study was not designed or powered to assess UGI bleeding outcomes and lacked a comparator arm, this interpretation remains hypothesis-generating and requires confirmation in adequately powered, controlled trials.

This study had certain limitations. First, although the GerdQ is a validated tool for diagnosing GERD and is a pragmatic choice in the acute stroke setting [13–15], GERD was not confirmed using objective diagnostic modalities such as endoscopy or pH monitoring. This reliance on symptom-based assessment may have reduced diagnostic precision. Second, selection bias is possible. Although we prospectively screened patients with AIS, many were unable to complete the GerdQ or had normal scores; therefore, only a small number of patients were ultimately included in the analysis. The small sample size and high exclusion rate underscore that this preliminary study should be interpreted with caution. Furthermore, the exclusion of patients unable to complete the questionnaire, who likely had more severe stroke and potentially more severe GERD symptoms [28,29], may have resulted in an overestimation of the observed benefit of fexuprazan. Third, the absence of a control or comparator group (e.g., PPI-treated patients) prevents meaningful comparison with standard therapy. However, this study still provides supportive preliminary observations that fexuprazan, which is already approved for GERD, could help prevent the aggravation of GERD symptoms in this specific population of patients with AIS treated with antithrombotics. Fourth, several potential confounders that may influence GERD symptoms, including dietary patterns, lifestyle factors, and concomitant medications such as NSAIDs, were not systematically assessed [30]. Finally, the 12-week follow-up period is relatively short to fully assess long-term safety and sustained gastrointestinal protection during chronic antithrombotic therapy. Future studies with longer follow-up durations are needed. Despite these limitations, this study had several strengths. We prospectively screened patients with AIS and implemented routine GerdQ assessments for all admitted patients as part of a standardized protocol conducted at baseline and 12 weeks. This approach enabled a structured and quantifiable evaluation of symptom changes over time. The ongoing PROTECT-HBR trial will provide definitive evidence regarding the role of P-CABs in patients with cardiovascular disease treated with antithrombotics.

In conclusion, this preliminary prospective single-arm study suggests that fexuprazan may be associated with improvement of GERD symptoms in Korean patients with AIS and GERD who are receiving antithrombotic therapy, as assessed by a structured symptom-based questionnaire. Given the very small sample size, absence of a comparator group, reliance on symptom-based assessment, and short follow-up duration, these findings should be interpreted strictly as hypothesis-generating. Larger controlled studies with objective outcome measures are required to confirm these observations and to define the role of P-CABs in this setting.

## Supporting information

S1 File Supporting figures and tables. This file contains S1 Fig (Flow diagram of patient inclusion) and S1 Table (GerdQ scores of individual participants).
(DOCX)
