# Supplementary material for: Fexuprazan for upper gastrointestinal protection in patients with ischemic stroke and gastroesophageal reflux disease (GERD) receiving antithrombotic therapy: A preliminary prospective single-arm observational Study
Source: PLoS One. 2026 Apr 3;21(4):e0346145. doi: 10.1371/journal.pone.0346145 (PMC13048479; doi:10.1371/journal.pone.0346145)
Supplement: S1 File — This file contains S1 Fig (Flow diagram of patient inclusion) and S1 Table (GerdQ scores of individual participants). (DOCX) [file pone.0346145.s001.docx]

**Fexuprazan for upper gastrointestinal protection in patients with ischemic stroke and gastroesophageal reflux disease (GERD) receiving antithrombotic therapy: A preliminary prospective single-arm observational Study**

**Running head:** Fexuprazan for upper gastrointestinal protection during antithrombotic therapy

Minyoul Baik, MD; JeongA Shin, MS; Joonsang Yoo, MD, PhD; and Jinkwon Kim, MD, PhD

Department of Neurology, Yongin Severance Hospital, Yonsei University College of Medicine, Yongin-si, Gyeonggi-do, South Korea

**S1 Fig. Flow diagram of patient inclusion**

**
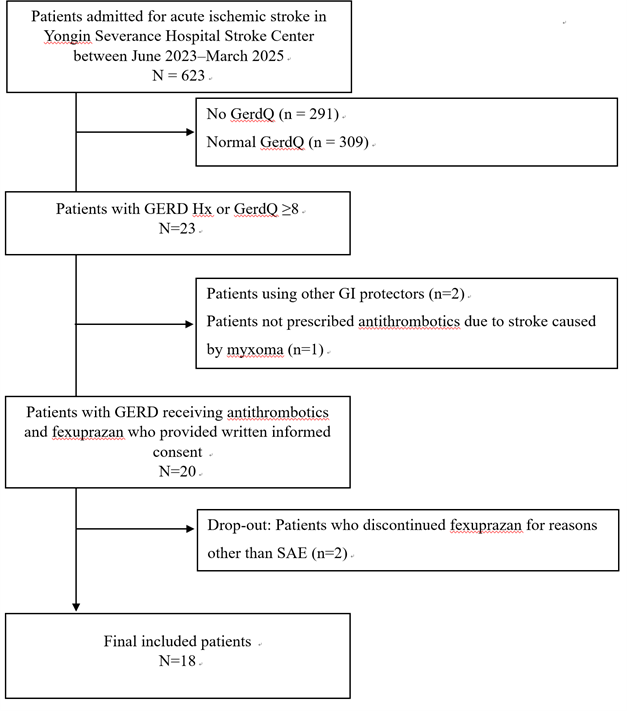
**

GERD, gastroesophagal reflux disease; GerdQ, Gastroesophageal Reflux Disease Questionnaire; GI, gastrointestinal; SAE, serious adverse event.

**S1 Table. GerdQ scores of individual participants**

|  |  | **Initial** | | | | | | | **12 weeks** | | | | | | |
| --- | --- | --- | --- | --- | --- | --- | --- | --- | --- | --- | --- | --- | --- | --- | --- |
| **Case** | **GERD** | **Total** | **Q1** | **Q2** | **Q3** | **Q4** | **Q5** | **Q6** | **Total** | **Q1** | **Q2** | **Q3** | **Q4** | **Q5** | **Q6** |
| **1** | + | 14 | 3 | 0 | 3 | 3 | 2 | 3 | 6 | 0 | 0 | 3 | 3 | 0 | 0 |
| **2** | + | 6 | 0 | 0 | 3 | 3 | 0 | 0 | 6 | 0 | 0 | 3 | 3 | 0 | 0 |
| **3** | + | 6 | 0 | 0 | 3 | 3 | 0 | 0 | 10 | 0 | 2 | 3 | 3 | 2 | 0 |
| **4** | + | 6 | 0 | 0 | 3 | 3 | 0 | 0 | 6 | 0 | 0 | 3 | 3 | 0 | 0 |
| **5** | + | 8 | 1 | 1 | 3 | 3 | 0 | 0 | 6 | 0 | 0 | 3 | 3 | 0 | 0 |
| **6** | + | 11 | 2 | 0 | 3 | 3 | 3 | 0 | 6 | 0 | 0 | 3 | 3 | 0 | 0 |
| **7** | + | 10 | 0 | 2 | 3 | 3 | 0 | 2 | 6 | 0 | 0 | 3 | 3 | 0 | 0 |
| **8** | - | 8 | 1 | 1 | 3 | 3 | 0 | 0 | 6 | 2 | 0 | 1 | 3 | 0 | 0 |
| **9** | + | 6 | 0 | 0 | 3 | 3 | 0 | 0 | 6 | 0 | 0 | 3 | 3 | 0 | 0 |
| **10** | + | 6 | 1 | 1 | 3 | 1 | 0 | 0 | 6 | 0 | 0 | 3 | 3 | 0 | 0 |
| **11** | - | 9 | 2 | 2 | 2 | 3 | 0 | 0 | 6 | 0 | 0 | 3 | 3 | 0 | 0 |
| **12** | - | 10 | 0 | 2 | 1 | 3 | 2 | 2 | 6 | 0 | 0 | 3 | 3 | 0 | 0 |
| **13** | + | 12 | 3 | 0 | 0 | 3 | 3 | 3 | 6 | 0 | 0 | 3 | 3 | 0 | 0 |
| **14** | - | 8 | 1 | 1 | 3 | 3 | 0 | 0 | 6 | 0 | 0 | 3 | 3 | 0 | 0 |
| **15** | - | 11 | 2 | 2 | 1 | 2 | 2 | 2 | 6 | 0 | 0 | 3 | 3 | 0 | 0 |
| **16** | - | 8 | 3 | 0 | 0 | 3 | 2 | 0 | 6 | 0 | 0 | 0 | 0 | 0 | 0 |
| **17** | - | 10 | 2 | 2 | 2 | 1 | 1 | 2 | 12 | 2 | 2 | 1 | 3 | 2 | 2 |
| **18** | - | 9 | 2 | 2 | 1 | 1 | 1 | 2 | 6 | 0 | 0 | 0 | 0 | 0 | 0 |

GERD, gastroesophagal reflux disease; GerdQ, Gastroesophageal Reflux Disease Questionnaire.
